# Application of the Updated WCRF/AICR Cancer Prevention Score as an Outcome for Cancer Survivors Participating in a Tailored and Intensive Dietary and Physical Activity Intervention

**DOI:** 10.3390/nu14224751

**Published:** 2022-11-10

**Authors:** Emily B. Hill, Elizabeth M. Grainger, Gregory S. Young, Steven K. Clinton, Colleen K. Spees

**Affiliations:** 1 Division of Medical Dietetics, School of Health and Rehabilitation Sciences, The Ohio State University College of Medicine, Columbus, OH 43210, USA; 2 The Ohio State University Comprehensive Cancer Center—James Cancer Hospital and Solove Research Institute, Columbus, OH 43210, USA; 3 Center for Biostatistics, The Ohio State University College of Medicine, Columbus, OH 43210, USA; 4 Division of Medical Oncology, Department of Internal Medicine, The Ohio State University College of Medicine, Columbus, OH 43210, USA

**Keywords:** lifestyle intervention, adherence, cancer survivorship, dietary patterns, physical activity patterns

## Abstract

The World Cancer Research Fund/American Institute for Cancer Research (WCRF/AICR) has defined evidence-based guidelines for cancer prevention. These recommendations have been operationalized into a quantitative index for individual assessment. Survivors of cancer are increasingly desiring guidance for diet and lifestyle, and in the absence of research in survivors, are often instructed to follow cancer prevention and public health guidelines. In this study, we examine the utility of the quantitative updated WCRF/AICR scoring criteria to assess change among cancer survivors with overweight/obesity (OW/OB) following an intensive behavioral intervention. We applied the WCRF/AICR scoring criteria (range 0–7) to examine changes over the duration of the study by paired *t*-tests. Two cancer survivor cohorts with OW/OB (*n* = 91) completed a six-month phase II clinical trial designed to improve dietary and physical activity patterns. At enrollment and post-intervention, participants completed assessments including anthropometrics, food frequency questionnaires, and objective evaluation of physical activity. Participants improved adherence to all scored recommendations, with a significant increase in mean score from enrollment (3.22 ± 1.06) to post-intervention (4.28 ± 1.04) (*p* < 0.001). Mean BMI and waist circumference improved (both *p* < 0.001). The greatest improvements were noted for fruit and non-starchy vegetable intakes (+39%, *p* < 0.001); the greatest decreases were observed for processed meat consumption (−70%, *p* < 0.001). The updated WCRF/AICR Score can be applied to cancer survivor intervention studies and provides a tool to compare trials in regard to the baseline status of populations enrolled and the success of the intervention. Future interventions incorporating standardized assessments will help guide effective strategies to improve the health and quality of life for cancer survivors.

## 1. Introduction

The overall 5-year relative survival rate for all cancers is 68% in the United States (USA), and many cancer survivors now live more than 10 years beyond diagnosis, necessitating an improved focus on healthy survivorship [1,2]. Cancer survivors are at increased risk of recurrence, second malignancies, and chronic disease. Personalized strategies are needed for risk assessment, intervention, and clinical surveillance to reduce disease burden and promote health [3]. While the cancer death rate has declined by over 30% in the last 30 years [2], the number of survivors with comorbid conditions, such as hypertension, diabetes, and obesity, has increased substantially [4]. Though many factors are involved in the etiology of these adverse health conditions, there has been growing interest in the contribution of modifiable lifestyle behaviors, including dietary and physical activity patterns. Further, the continued expansion of precision medicine initiatives highlights the importance of designing interventions tailored to the unique underlying characteristics and needs of the population of interest in an effort to improve health outcomes [5].

Global public health recommendations focusing on diet, energy balance, and fitness for cancer prevention have been defined by several organizations, including the World Cancer Research Fund/American Institute for Cancer Research (WCRF/AICR) and the American Cancer Society (ACS). These guidelines are based on a rigorous review of the scientific literature to provide guidance for behavior modification in an effort to reduce cancer risk [6,7]. The ACS, the National Comprehensive Cancer Network (NCCN), and the American Society of Clinical Oncology (ASCO) similarly have published guidelines for survivors and to assist clinicians in providing evidence-based recommendations to improve health and reduce risk of morbidity and mortality among survivors [8,9,10]. Unfortunately, the profound lack of prospective clinical trials in cancer survivors means that global cancer prevention guidelines or national guidelines are typically the foundation of advice for cancer survivors. In sum, cancer survivors are advised on the maintenance of healthy body weight, adequate physical activity, and consumption of a diverse and primarily plant-based dietary pattern. The 2018 WCRF/AICR Third Expert Report, *Diet, Nutrition, Physical Activity, and Cancer: A Global Perspective*, highlights the potential benefit of considering these factors together as a pattern of behavior rather than assessing each in isolation [6,11].

The limited research available demonstrates that cancer survivors’ compliance to these guidelines is associated with improved health-related quality of life, including increases in physical functioning and reductions in fatigue [12,13]. Consuming higher quality dietary patterns among cancer survivors is associated with both cancer-specific and all-cause mortality [14,15,16,17,18]. Despite this evidence, most cancer survivors fail to meet the basic recommendations, and many remain overweight or obese and exhibit inadequate dietary and physical activity patterns [19,20,21]. Comprehensive, multicomponent, theory-based lifestyle interventions focusing on nutrition and exercise behaviors within this population have been established to address this gap [22,23,24]. Participation in such programs leads to positive effects on not only diet and physical activity patterns, but also on health outcomes related to cardiometabolic risk and quality of life of cancer survivors [25,26,27]. Optimizing and personalizing the intervention methods as well as the intensity of effort needed to achieve meaningful changes in diet and lifestyle are critically needed to establish programs integrated into cancer care.

Based upon the updated recommendations within the Third Expert Report, a collaborative group consisting of individuals from WCRF International, AICR, the National Cancer Institute (NCI) at the National Institutes of Health, and ISGlobal developed a standardized scoring approach for measuring adherence that was reviewed and approved by the WCRF/AICR Continuous Update Project (CUP) Expert Panel [28]. This work culminated in the first standardized criteria for computing a total adherence score to aid in studying how compliance with the updated WCRF/AICR Cancer Prevention Recommendations may impact health. In a follow-up commentary, this group provided additional guidance for the interpretation and highlighted a specific interest in the application of the score within a population of cancer survivors [29].

To date, the application of the standardized 2018 WCRF/AICR Score has primarily been employed in large prospective cohorts or within case–control studies to determine the association between the score and cancer risk at the population level [30,31,32,33,34]. While few studies have investigated the relationship between the 2018 WCRF/AICR Score and health and quality of life outcomes among cancer survivors [12,14,35], to our knowledge, no evaluation of change within the context of a lifestyle intervention to determine the impact of comprehensive behavior modification programming on cancer survivor adherence has been conducted using these updated criteria. The purpose of the present study was therefore to assess change in score after participation in a tailored and intensive dietary and physical activity intervention.

## 2. Materials and Methods

This study employed secondary analyses of pooled data from two independent 6-month intensive dietary and physical activity interventions for cancer survivors with overweight and obesity (OW/OB, *n* = 91). Both studies were designed with the aim to help survivors improve adherence to the WCRF/AICR Cancer Prevention Recommendations and included the same intervention components, as described below.

### 2.1. Participants

Two cohorts of individuals were recruited from The Ohio State University Comprehensive Cancer Center—James Cancer Hospital and Solove Research Institute, outpatient oncology clinics, community-based survivorship programming, and online via email and social media outlets targeting cancer survivors. Recruitment fliers were also distributed in person and via email. Interested participants were asked to complete an online screener to confirm eligibility.

Inclusion criteria included: (1) voluntary agreement to participate and sign an informed consent; (2) ability to speak English; (3) age ≥ 18 years; (4) completion of cancer treatment (e.g., chemotherapy, radiation therapy, and/or surgery) within the previous 48 months (use of adjuvant hormone therapy was permissible); (5) no current evidence of active cancer; and (6) body mass index (BMI) ≥ 25 kg/m^2^. Exclusion criteria included: (1) cognitive inability to consent; (2) physical or mental condition precluding participation in the intervention; (3) previous participation in our feasibility pilot or recent participation in another diet or physical activity program [25]; (4) prescription of insulin, oral hypoglycemic, or lipid-lowering medication within the previous 3 months; (5) consumption of medication with contraindication to increased intakes of fruits and vegetables; (6) refusal to refrain from consumption of non-prescription nutritional supplements; and (7) diagnosis of active metabolic or digestive illness, renal or hepatic insufficiency, cachexia, short bowel syndrome, or pregnancy.

Upon confirmation of eligibility, participants completed written informed consent for study participation, HIPAA Authorization Forms, were scheduled for a study orientation, and were registered with the Clinical Trials Office. The studies were approved by The Ohio State University Clinical Scientific Review Committee and the Institutional Review Board and are registered at https://clinicaltrials.gov/ (accessed on 12 October 2022) (NCT02268188 and NCT03489213).

### 2.2. Intensive Behavioral Intervention

Participants in both cohorts completed a similar 6-month intensive dietary and physical activity intervention. Details regarding intervention components have been previously published [36]. Throughout, participants were provided: (1) weekly produce harvesting at an urban garden; (2) semi-monthly group education, behavioral self-management, and skill-development sessions; (3) individualized remote nutrition counseling informed by motivational interviewing with a trained registered dietitian; (4) supportive technologies including a fitness tracker for personal activity monitoring; and (5) access to a secure website for additional information. The primary objective of the intervention was to improve adherence to the dietary and physical activity patterns recommended by the WCRF/AICR [6]. These recommendations also align with other public health guidelines, including those set forth by the ACS, the U.S. Dietary Guidelines for Americans (DGA), and the Physical Activity Guidelines for Americans [7,37,38].

The dietary component of the intervention encouraged a plant-based dietary pattern through increased intakes of fruits and vegetables to meet recommendations of 400 g of fruits and non-starchy vegetables and 30 g of fiber per day. Additional dietary recommendations included increased consumption of whole grains and plant-based proteins with moderate consumption of low-fat dairy, lean meats, and heart-healthy fats and oils. Participants were encouraged to limit red meat intakes to 12 to 18 ounces (approximately 350–500 g) per week and to avoid processed meats, sugar-sweetened beverages, and alcohol. Similarly, they were asked to reduce consumption of ultra-processed foods, defined by the WCRF/AICR guidelines as “fast foods” and other processed foods high in fat, starches, or sugars. They were additionally encouraged to increase moderate-to-vigorous physical activity, which was prescribed primarily as walking with a modest goal of increasing total steps per day toward achievement of 10,000 daily steps. As all participants had OW/OB at study enrollment, weight loss was also promoted over the course of the intervention.

### 2.3. Data Collection

#### 2.3.1. Clinical Assessment Visits

Participants completed anthropometric measurements, dietary and physical activity assessments, lifestyle questionnaires, and clinical evaluations at enrollment (month 0) and post-intervention (month 6). Clinical assessment visits were scheduled between 7:00 a.m. and 10:00 a.m. to control for diurnal variation. Participants were asked to refrain from vigorous exercise and alcohol consumption for 72 h prior and to adhere to a 12-h fast prior to scheduled visits. Secure online surveys were used to collect sociodemographic information, medical history, and lifestyle behaviors including dietary patterns. All data were collected and stored in REDCap (Research Electronic Data Capture, Vanderbilt, Nashville, TN, USA) data capture tools hosted at The Ohio State University [39].

#### 2.3.2. Dietary Patterns

Dietary intake was assessed via the VioScreen (Viocare, Inc., Princeton, NJ, USA) 30-day food frequency questionnaire (FFQ). This validated FFQ utilizes computer software to graphically depict foods and portion sizes for more accurate estimates of food and beverage consumption patterns [40]. The VioScreen FFQ is based on paper FFQs developed at the Fred Hutchinson Cancer Research Center and utilizes Nutrition Data System for Research (NDSR-V45) developed by the Nutrition Coordinating Center (NCC) at the University of Minnesota for dietary analysis [41].

#### 2.3.3. Physical Activity Patterns

In the first cohort, hip pedometers (Omron Healthcare Co., Inc., Lake Forest, IL, USA) were provided at enrollment clinic visits to serve as both a method of behavioral reinforcement and for data collection. Participants were asked to wear the device at the hip each day for the duration of the 6-month intervention and to submit daily steps via a secure online form at least once weekly. Daily step counts from valid wear days in the week following enrollment data collection and the week prior to post-intervention data collection were used to determine the mean step count. As participant wear time and cadence data were unavailable, valid days were determined as those with a minimum of 100 steps/day and no more than 50,000 steps/day recorded [42]. In the second cohort, a Fitbit Charge 2 (Fitbit Inc., San Francisco, CA, USA) was provided to all participants at enrollment clinical assessment visits. Participants were asked to wear the Fitbit on their nondominant wrist each day for the duration of their participation in the study, syncing the device to the Fitbit server via Bluetooth at minimum of once per week. Step count data were exported monthly, and daily step counts from valid wear days were similarly tabulated for enrollment and post-intervention.

#### 2.3.4. Anthropometric and Clinical Measures

Participant height and weight were measured using standard protocols with a fixed stadiometer (Health-o-Meter Professional Products, Pelstar LLC, Bridgeview, IL, USA in the first cohort, Holtain Limited, Crymych, Dyfed, UK in the second cohort) and a calibrated Pro Plus digital scale (Health-o-Meter Professional Products, Pelstar LLC, Bridgeview, IL, USA), respectively. Height was recorded to the nearest 0.1 cm, and weight to the nearest 0.1 kg. Waist circumference (WC) was measured in triplicate between the costal margin and iliac crest to the nearest 1 mm. Mean WC was used for analyses.

### 2.4. Calculation of WCRF/AICR Score

Adherence to WCRF/AICR Cancer Prevention Recommendations was scored utilizing a standardized scoring system [28]. Briefly, seven components representing operationalization of the recommendations were scored according to predefined quantitative cutoffs to generate a total score ranging from 0 to 7 points, with a higher score indicative of greater compliance with recommendations. A score of 1 point was assigned for each component when a recommendation-specific cutoff was met, with 0.5 points when partially met, and 0 points when not met. For two recommendations, components were split between two criteria, and 0.5 points were assigned when a sub-recommendation was met, 0.25 when partially met, and 0 points when not met. Scores for sub-recommendations were summed to generate a total score for the recommendation. The scoring of each component is detailed in Table 1.

### 2.5. Adaptation of Scoring

For many components, data were collected and could be scored as recommended. For others, it was necessary to adapt scoring criteria slightly based on data availability. Due to the provision of pedometers/Fitbits for data collection, daily step counts were employed to determine adherence to the physical activity component of the recommendations. Evidence supports the translation of daily step counts to minutes of moderate-to-vigorous physical activity (MVPA), with a volume of 7000 steps equivalent to 150 min per week among older adults and special populations, including cancer survivors [45,46]. Similarly, dietary intakes of fruits and non-starchy vegetables were determined using Food Patterns Equivalents Database (FPED) groupings and reported in cup equivalents per day. Intakes of five 80-g servings or 2.5 cup equivalents of fruits and non-starchy vegetables per day are equivalent to 400 g [47]. Details regarding adaptation of scoring criteria for these components are included in Table 1, with additional detail regarding categorization of ultra-processed foods for assessment of “fast food” and other processed food intakes in Supplementary Table S1.

### 2.6. Statistical Analysis

Data analyses were conducted in collaboration with a biostatistician at The Ohio State University Center for Biostatistics. Descriptive statistics were generated for all demographic and outcome measurements using SAS v9.4 (SAS Institute, Cary, NC, USA). Additionally, statistical analyses for the effect of the intervention on dietary and clinical measures were conducted by comparing pre- and post-intervention scores for pooled cohort data. The hypothesis of no change in these variables was tested using a paired *t*-test. For variables exhibiting many zero-values and skewed distribution, a non-parametric Wilcoxon signed rank test was employed. Significance was established a priori α = 0.05. Some variables were log-transformed prior to analysis due to heteroscedasticity. For these variables, pre- and post-intervention differences are expressed as fold change. All other values are reported on the original scale.

## 3. Results

### 3.1. Participants

A total of 119 individuals across both cohorts were enrolled. Among these, four participants withdrew prior to baseline assessments, six were removed from the study due to cancer recurrence, and 18 withdrew due to non-study-related health concerns or personal issues such as time conflicts. Among those who completed the intervention, participants were primarily female and White/Caucasian, with a mean age of 53.0 ± 11.5 years (Table 2). Predominant cancers included breast (54.9%), leukemia/lymphoma (11.0%), and female reproductive (9.9%). Compliance with the intervention was high among both cohorts, with mean group education attendance of 87%, and 76 of 91 participants (84%) attending at least 75% of offered sessions, as assessed by in-person attendance tracking at education sessions. All participants regularly harvested produce over the course of the intervention, and a substantial proportion of individuals used remote nutrition counseling (77%) and the secure study website (92%) for additional support. Of those who completed the intervention, all participants attended in-person clinic visits for clinical and dietary assessments at enrollment and post-intervention, while 90 of 91 individuals provided physical activity data from fitness trackers.

### 3.2. Changes in Lifestyle Behaviors

Significant reductions in mean BMI and waist circumference as well as dietary intakes of ultra-processed foods, red meat, and processed meat were noted (all *p* < 0.001, Table 3). Greatest increases in dietary intakes were noted for fruits and non-starchy vegetables, which increased by nearly 1.5 servings/day (*p* < 0.001). Greatest decreases were observed for processed meat intakes, which were reduced from 107 to 36 g/week. For sugar-sweetened beverages and alcohol, median intakes remained close to zero and a negative shift in data was demonstrated, indicating a significant decrease from enrollment to post-intervention (*p* = 0.008 and *p* = 0.005, respectively). Participants also increased physical activity by over 1100 steps/day (*p* < 0.001) over the course of the intervention.

### 3.3. Changes in WCRF/AICR Score

**Figure 1:**
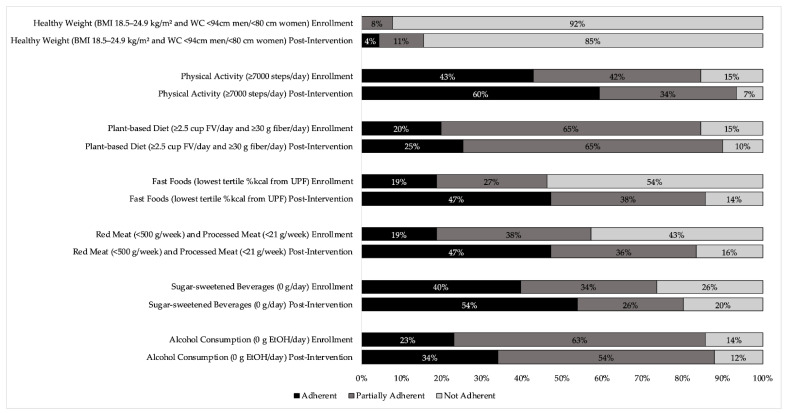
Change in adherence to WCRF/AICR recommendations over cancer survivor participation in an intensive behavioral intervention focusing on dietary and physical activity patterns. Participants’ mean score for each component of the WCRF/AICR Recommendations for Cancer Prevention was categorized as “Adherent” (score of 1; meeting recommendation), “Partially Adherent” (score between 0 and 1; partially meeting recommendation), and “Not Adherent” (score of 0; not meeting recommendation) at enrollment and post-intervention. Mean scores for components increased over time, and the proportion of individuals who were adherent to each recommendation likewise increased from enrollment to post-intervention, demonstrated by a shift toward darker bars at post-intervention; BMI body mass index, WC waist circumference, FV fruits and vegetables, UPF ultra-processed foods, EtOH ethanol.

The total score at enrollment was 3.22 ± 1.06 and was increased by 33% to 4.28 ± 1.04 at post-intervention (*p* < 0.001), with participants significantly improving adherence to all recommendations. At enrollment, the lowest mean component score was displayed for a healthy weight, as no participant began the study adherent to this recommendation. Greatest adherence was observed for the plant-based diet component, though only 20% of participants were meeting this recommendation at enrollment. Healthy weight remained the recommendation with the poorest adherence at post-intervention, while mean physical activity adherence was highest, with 93% of individuals fully or partially meeting the recommendation after the intervention. The proportion of individuals adherent, partially adherent, or not adherent to each recommendation at each time point is visualized in Figure 1.

## 4. Discussion

Adherence to recommendations for modifiable behaviors is variable among intervention strategies and among individuals on trials and is often inconsistently defined or quantified [28,29]. This leads to a lack of standardization for evaluating outcomes among survivors on diet and lifestyle trials, which impedes the ability to translate research findings into clinical practice. This study directly addresses this issue by incorporating the 2018 WCRF/AICR Score within a lifestyle intervention to quantify a change between enrollment and post-intervention. One value of the WCRF/AICR Score is that it represents an integrative measure of components that together may have a greater impact than individual variables. Results demonstrate the feasibility of employing the WCRF/AICR Score in studies where accurate food intake has been measured. Behavioral components driving the total score were sensitive to the changes reported at the individual level, exhibiting clinical relevance and proof of principle for the application of the score as a simple metric of integrated behavioral outcomes in survivorship research. Hence, uniform methodological application of the WCRF/AICR criteria allows for greater ease in the interpretation of interventions and comparisons across clinical trials so as to better inform effective interventions tailored to the needs of specific survivorship groups.

Participants in this study significantly improved adherence to the seven measured recommendations within our intervention, demonstrated by a mean increase of over one point in the total score. These results are among the first to provide such an evaluation, as there is a paucity of research investigating the impact of comprehensive lifestyle interventions on integrated changes in behavior using such scoring mechanisms. In a recent analysis of a remotely delivered dietary and physical activity intervention for breast cancer survivors in Australia, small-to-moderate effects on adherence for several behaviors as measured by a composite score based on the 2007 WCRF/AICR guidelines were noted [48]. Similar to survivors in this study, authors reported <10% of women met more than four of seven recommendations at baseline, with significant, clinically meaningful, and durable improvements in overall adherence after the six-month intervention. Our study provides additional evidence using the updated, standardized criteria for 2018 WCRF/AICR scoring to further support the use of comprehensive lifestyle interventions to help survivors improve recommendation adherence.

Though a change in the updated 2018 WCRF/AICR Score has not previously been evaluated longitudinally in the context of a behavioral intervention for cancer survivors, among the general population, an increase in adherence to this scale has been associated with an approximately 10% lower relative risk of both all-cause and cancer-specific mortality [49]. Further, based upon analysis of adherence among survivors, those with the greatest adherence exhibit a 33% lower mortality rate compared to those with low adherence [16], indicating improvements in body weight, physical activity, and diet within our cohorts may have a substantial impact on subsequent health risk. Taken together, participant increases in fruit, vegetable, and fiber intakes with concomitant decreases in foods that are heavily processed and high in salt, sugar, and saturated fat lead to a marked improvement in adherence to the dietary recommendations set forth by WCRF/AICR, ACS, and other national guidelines such as the DGA. As most cancer survivors fall short of these recommendations, similar to the rest of the US population [50], findings support previous literature suggesting intensive behavioral intervention tailored to this population is foundational to addressing gaps.

Particularly striking were participant changes in intakes of highly processed foods, including a 70% reduction in intakes of processed meats. Based upon extensive review of the literature, the International Agency for Research on Cancer (IARC) has identified processed meat as a carcinogen, and the WCRF/AICR Cancer Prevention Recommendations emphasize limits on consumption [51]. Recent analyses have also indicated that processed, but not unprocessed, meat consumption is associated with both increased CVD and mortality risk [52]. Accordingly, reduction in processed meat intake was regularly encouraged during the nutrition education component of this study, and participants were taught how to identify processed meats and healthy substitutions. Post-intervention intakes of approximately 30 g per week are well below average US consumption, as data indicate mean intakes of approximately 71 g per day in men and 52 g per day in women [53]. In addition to reducing the risk of CVD, lowering processed meat intakes may also help individuals shift towards plant-based dietary patterns that are more environmentally sustainable than those high in meat [54,55]. The sustainability of the food system to support health-promoting dietary patterns is an important consideration for public health guidance highlighted in the Scientific Report of the 2020 U.S. Dietary Guidelines Advisory Committee, and individuals shifting dietary patterns to better align with WCRF/AICR recommendations is one way to achieve this goal [56].

For the “fast foods” component of the total score, ultra-processed food intakes are not assessed based on adherence to a specific value or range but rather upon tertile of intake within the population. Scores assigned based upon ultra-processed food intakes of participants within this study therefore may not be representative of those displayed in the US population. Indeed, while our participants reported approximately 45% of calories from ultra-processed food at enrollment, data suggests nearly 60% of calories in the US come from ultra-processed foods [57,58]. Current evidence indicates ultra-processed food consumption may be associated with poor diet quality, excess body weight, and increased risk for cardiometabolic disease as well as all-cause mortality [59,60,61]. Given the lower baseline intakes of ultra-processed foods in our cohort compared to the general population, a reduction of greater than 10% is substantial. Analyses from the NutriNet-Santé prospective cohort indicate this magnitude of change may lead to significant reductions in risk for CVD [62]. This is particularly important in the context of cancer survivorship and suggests reduction in fast foods, frozen and pre-packaged meals high in sodium and saturated fat, and other foods with high levels of industrial processing as recommended by WCRF/AICR may be useful for improving diet quality and reducing risk for excess weight and other comorbidities in addition to cancer.

Individuals significantly increased their consumption of fruits and vegetables to over five cup equivalents per day, with 75% of study participants partially or fully meeting recommendations post-intervention. This increase in consumption was both encouraged and expected, as participants in this study were given access to free, fresh produce for the duration of the six-month intervention, including foods such as melons, berries, peppers, squash, tomatoes, leafy greens, cabbage, and broccoli. Increases in fruit and vegetable consumption are commonly reported in other clinical trials focusing on weight loss and dietary patterns among survivors, as adequate intake of fruits and vegetables is consistently a key nutrition recommendation [63,64].

Evaluation of adherence is difficult, as behaviors are dynamic and assessment methods prone to error. However, advances in technology have led to dietary assessment tools designed to reduce participant and researcher burden and accompanying biases. Commonly, FFQs now employ online delivery allowing for self-administration, application of complex skip-patterns, photographs of standard portions, and/or automated coding and analysis for calculation of intakes [65,66]. The combination of different dietary assessment methods has also been proposed as a means by which to address methodological limitations [67,68,69]. With rapid advancements in dietary assessment methodology and promising biomarkers of food intake currently under study [70], it may soon be possible to include objective indicators of dietary factors, such as fruits and vegetables [71], sugar-sweetened beverages [72,73,74] or whole grains [75,76,77] to complement self-report data when assessing adherence to the dietary guidance within the WCRF/AICR Cancer Prevention Recommendations.

In previous studies employing the 2018 WCRF/AICR Score, physical activity was defined based on occupation and/or self-reported leisure-time physical activity [12,14,30,31,32,33,34,35]. As the literature suggests that cancer survivors may significantly overestimate MVPA compared to activity determined by objective measurement, we adapted scoring criteria for the physical activity component of the score to employ data collected from fitness trackers [78,79]. In addition, while participants were encouraged to increase steps to 10,000 steps/day, which is on the higher end of the recommended range, previous research indicates approximately 7000 steps/day provides equivalent levels of MVPA in this special population due to inherent characteristics that may limit mobility and/or physical endurance [46]. Further, a smaller effect size and a mean increase ranging from 800–2000 steps/day have been observed among older adults and special populations after pedometer-based physical activity interventions when compared to younger, healthy adults [46]. Thus, while mean step counts did not reach the prescribed goal, observed improvements align with other studies and reflect an expected increase in adherence to the guidelines. Providing clear methods for the application of step data in the calculation of this component while considering the distinct differences cancer survivors may exhibit when compared to the general population fills an important gap in the literature. Further, the use of commercially available trackers, such as hip pedometers or wearable Fitbits, allows for methods to be applied in a clinical setting where self-report physical activity assessments may not be standard of care [80,81]. Recent data indicate approximately one in five US adults use a fitness tracker [82]. As such, healthcare providers working with survivors can employ these devices to measure physical activity in tandem with other measures of lifestyle behaviors.

Dietary, physical activity, and clinical data were integral parts of the remote nutrition counseling component of the intervention. Individual- and deidentified group-level data were shared with participants through tailored reports over the duration of the study to assist with goal setting as well as to provide a source of reinforcement and motivation [83]. Feedback related to dietary intakes allows individuals to analyze their behaviors and track trends over time, leading to increased awareness of patterns and practices as they initiate behavior change [84]. Similarly, the literature demonstrates fitness trackers used within interventions for cancer survivors can lead to reductions in sedentary behaviors and improved step counts and other measures of physical activity, in part due to their ability to help individuals develop and implement self-monitoring skills [85]. We foresee the increased incorporation of systematic dietary, physical activity, and biological assessment into survivorship care planning to provide integrated physiological and lifestyle data, leading to the optimization of diet and exercise recommendations tailored at the individual level for the delivery of future interventions.

Individuals demonstrated a mean reduction in BMI of >1 kg/m^2^ from enrollment to post-intervention. Though participants significantly improved weight status, these improvements were not of sufficient magnitude to move individuals across waist circumference cut points or BMI categories and therefore were not reflected by a substantial improvement in adherence. Further study is warranted to investigate metrics that may provide greater clinical utility when monitoring change in weight within a behavioral intervention to define improvement. Assigning scores based upon clinically meaningful reductions in weight (e.g., nonadherence with 0% weight loss or weight gain, partial adherence with 0–<5% loss, full adherence with ≥5% loss) may provide greater insight into the efficacy of intervention [86]. In addition, longitudinal follow-up allowing for more observations and assessment for the development of conditions such as hypercholesterolemia or hypertension may be used to test the predictive ability of changes in WCRF/AICR Score on these outcomes. Healthcare providers should consider these factors if they wish to apply the score to evaluate the impact of lifestyle change in clinical settings.

This study has many strengths, including evaluation of a tailored and intensive dietary and physical activity intervention with a focus on cancer survivors, data collection at multiple time points, and application of the updated standardized WCRF/AICR scoring criteria to determine the change in adherence after participation. However, it is not without limitations. The current WCRF/AICR Score provides equal weighting for all components, though evidence suggests differential effects exist, and the score does not address all major risk factors associated with cancer-related outcomes [28,29]. Further, while some demographics reflect the general population, the participants in the present study were relatively homogenous, as >90% were Caucasian and female, with a majority of participants being diagnosed with breast cancer, perhaps limiting the generalizability of results. Thus, findings should be replicated in a larger, more diverse population. Similarly, without the inclusion of a control group for comparison, we relied on pre–post changes in individual participants. While significant improvements in both dietary intakes and objective clinical variables such as weight and waist circumference were documented, this study design introduces some errors not seen in randomized controlled trials, and thus, findings may lack external validity. Regarding the nature of the dietary intervention, which was not a highly controlled feeding study, it may be difficult to elucidate small changes in dietary intakes due to limitations in self-report dietary recall methods such as recall bias, though these were minimized by the use of a validated graphical FFQ. Lastly, the six-month duration of the intervention does not allow for evaluation of the sustainability of behavior change nor association with disease endpoints such as cancer recurrence or mortality. Larger trials with extended longitudinal follow-up are warranted.

## 5. Conclusions

The use of the WCRF/AICR standardized scoring criteria for assessing baseline compliance to recommendations and changes following an intervention adds significant value in survivorship research. The overall WCRF/AICR Score provides an integrated measure of diet quality, physical activity, and body weight for cancer survivors. The WCRF/AICR Score is also a useful measure to quantitatively assess the impact of modifiable lifestyle interventions and to compare success relative to the intensity and types of intervention studies in various phase I, II, and III trials. Moving forward, such efforts are key to providing cancer survivors undergoing diverse treatments with more personal and precise guidelines to promote longevity, health, and quality of life.

## Figures and Tables

**Table 1:** Operationalization and Adaptation of Standardized Scoring of WCRF/AICR recommendations for survivors of cancer participating in an intensive behavioral intervention focusing on dietary and physical activity patterns.

2018 WCRF/AICR Recommendations	Operationalization of Recommendations [28]	Adaptation	Points
1. Be a healthy weight	BMI (kg/m^2^)	None	
	18.5–24.9		0.5
	25–29.9		0.25
	<18.5 or ≥30		0
	Waist circumference (cm)	None	
	Men: <94		0.5
	Women: <80		
	Men: 94–<102		0.25
	Women: 80–<88		
	Men: ≥102		0
	Women: ≥88		
2. Be physically active	Total moderate-vigorous physical activity (minutes/week)	Total steps (steps/day)	
	≥150	≥7000	1
	75–<150	3500–6999	0.5
	<75	<3500	0
3. Eat a diet rich in whole grains, vegetables, fruit, and beans	Fruits and vegetables (g/day)	Fruits and vegetables (cup eq/day)	
	≥400	≥2.5	0.5
	200–<400	1.25–2.5	0.25
	<200	<1.25	0
	Total fiber (g/day)	None	
	≥30		0.5
	15–<30		0.25
	<15		0
4. Limit consumption of “fast foods” and other processed foods high in fat, starches, or sugars	Percent of total kcal from ultra-processed foods (UPFs) ^a^	None	
	Tertile 1		1
	Tertile 2		0.5
	Tertile 3		0
5. Limit consumption of red and processed meat	Total red meat (g/week) and processed meat (g/week)	None	
	Red meat <500 and processed meat <21		1
	Red meat <500 and processed meat 21–<100		0.5
	Red meat >500 or processed meat ≥100		0
6. Limit consumption of sugar-sweetened drinks	Total sugar-sweetened drinks (g/day)	None	
	0		1
	>0–≤250		0.5
	>250		0
7. Limit alcohol consumption	Total ethanol (g/day)	None	
	0		1
	Men: >0–≤28		0.5
	Women: >0–≤14		
	Men: >28		0
	Women: >14		
**Total WCRF/AICR Score Range**			**0–7**

^a^ UPF: Ultra-processed food list determined for all foods based on NOVA classification [43,44] and in collaboration with NIH/NCI, WCRF/AICR, and ISGlobal. Available as Supplementary Table S1.

**Table 2:** Participant characteristics of survivors of cancer participating in an intensive behavioral intervention focusing on dietary and physical activity patterns (*n* = 91).

Participant Characteristic		*n* (%)
Age, years (mean ± SD)		53.0 ± 11.5
Sex	Female	82 (90.1)
	Male	9 (9.9)
Race/Ethnicity	White/Caucasian	82 (90.1)
	Black/African American	7 (7.7)
	Asian	2 (2.2)
Marital Status	Married	54 (59.3)
	Never Married	19 (20.9)
	Divorced	12 (13.2)
	Other ^a^	6 (6.6)
Education	Grade 12 Equivalent	7 (7.7)
	College 1 to 3 years	11 (12.1)
	College 4 years or more	38 (41.8)
	Professional or Graduate	35 (38.5)
Employment	Employed or Self-employed	68 (74.7)
	Retired	21 (23.1)
	Other ^b^	2 (2.2)
Household Income	USD 10,000–24,999	6 (6.6)
	USD 25,000–49,000	9 (9.9)
	USD 50,000–74,999	5 (5.5)
	≥USD 75,000	28 (30.8)
	Do not know/Prefer not to answer	43 (47.3)
Primary Cancer	Breast	50 (54.9)
	Leukemia/Lymphoma	10 (11.0)
	Ovarian/Uterine/Cervical/Endometrial	9 (9.9)
	Thyroid	6 (6.6)
	Prostate	5 (5.5)
	Oral/Head/Neck	4 (4.4)
	Colorectal	2 (2.2)
	Other ^c^	5 (5.5)

^a^ Includes widowed, member of an unmarried couple, and prefer not to answer. ^b^ Includes unable to work and out of work < 1 year. ^c^ Includes brain, melanoma/skin, and pancreatic.

**Table 3:** Adherence to lifestyle behaviors comprising WCRF/AICR Score by survivors of cancer participating in an intensive behavioral intervention focusing on dietary and physical activity patterns (*n* = 91).

WCRF/AICR Component	(Mean ± SD)		Difference (95% CI)	*p*-Value
	Enrollment	Post-Intervention		
Healthy weight				
BMI (kg/m^2^)	32.7 ± 4.6	31.5 ± 4.8	−1.1 (−1.4, −0.8)	<0.001
Waist circumference (cm)	105.4 ± 11.9	103.0 ± 13.0	−2.4 (−3.6, −1.1)	<0.001
Physical activity (steps/day) ^a^	6958 ± 3258	8066 ± 3215	1107 (522, 1693)	<0.001
Plant-based diet				
Fruits and non-starchy vegetables (cup eq/day) ^b^	3.7 ± 1.8	5.1 ± 2.3	1.39 (1.27, 1.52)	<0.001
Fiber (g/day) ^b^	23.7 ± 11.6	25.6 ± 10.1	1.11 (1.03, 1.19)	0.007
Fast foods (% kcal from UPF/day)	45.1 ± 14.0	34.6 ± 11.4	−10.5 (−13.5, −7.5)	<0.001
Red meat (g/week) ^b^	258 ± 206	162 ± 142	0.62 (0.48, 0.81)	<0.001
Processed meat (g/week) ^b^	107 ± 130	36 ± 43	0.30 (0.21, 0.42)	<0.001
Sugar-sweetened beverages (g/day) ^c^	228.8 ± 398.5	161.9 ± 345.0	0.0 (−82.7, 0.0)	0.008
Alcohol (g/day) ^c^	6.7 ± 12.4	5.4 ± 11.0	0.0 (−1.5, 0.0)	0.005

^a^*n* = 90 due to missing fitness tracker data. ^b^ Data log-transformed for analyses and difference expressed as fold change. ^c^ Difference expressed as median change (25% ile, 75% ile) and evaluated using non-parametric Wilcoxon signed rank due to large number of zero values and skewed distribution. BMI body mass index, UPF ultra-processed foods.

## Data Availability

The data presented in this study are available on request from the corresponding author.

## Supplementary Materials

The following supporting information can be downloaded at: https://www.mdpi.com/article/10.3390/nu14224751/s1, Table S1: Ultra-processed Foods (UPF) List.
