# Supplementary material for: Application of the Updated WCRF/AICR Cancer Prevention Score as an Outcome for Cancer Survivors Participating in a Tailored and Intensive Dietary and Physical Activity Intervention
Source: Nutrients. 2022 Nov 10;14(22):4751. doi: 10.3390/nu14224751 (PMC9699073; doi:10.3390/nu14224751)
Supplement: Supplementary file 1 [file nutrients-14-04751-s001.zip › nutrients-1996502-supplementary.pdf]

Supplementary Table S1. Ultra-processed Foods (UPF) List

**FFQ foods included in UPF food list**
All other cheese, such as American, cheddar or cream cheese, including cheese used in cooking
Asian-style (stir-fried) noodles and rice, such as chow mein, fried rice and pad Thai
Asian-style noodle soup
Burritos, tacos, tostadas and quesadillas
Buttered or regular microwave popcorn
Canned tuna, tuna salad and tuna casserole
Cheese sauce and cream sauce
Chocolate, candy bars, and toffee
Coleslaw
Cooked standard grain cereals
Cookies and cakes - lowfat
Cookies and cakes - regular
Corn tortillas
Cornbread and corn muffins
Cream soups such as chowders, potato and cheese
Cream, non-dairy liquid (Coffee)
Cream, non-dairy liquid (Milk in cooked cereal)
Cream, non-dairy liquid (Milk on cold cereal)
Cream, non-dairy liquid (Milk on cooked cereal)
Cream, non-dairy liquid (Tea)
Diet soft drinks
Doughnuts, pies and pastries
Enchiladas and tamales
Flour tortillas
French fries, fried potatoes and hash browns
Fried chicken, including chicken nuggets and tenders
Fried fish, fish sandwich and fried shellfish (shrimp, oysters)
Granola bars and cereal bars such as Nutri-Grain Bars
Greek yogurt, sweetened or with fruit, full-fat
Greek yogurt, sweetened or with fruit, lowfat
Greek yogurt, sweetened or with fruit, nonfat
Highly fortified cereals
Ice cream and milkshakes
Jam, jelly, honey, syrup or sugar on bread, pancakes or waffles
Japanese Noodles
Ketchup
Low fat cheese and low fat cream cheese, including cheese used in cooking
Low fat sour cream (Fats on potatoes, rice, noodles and beans)
Low fat sour cream (Fats used on vegetables)
Low or nonfat crackers, such as saltines and SnackWell's

Low or nonfat frozen desserts such as lowfat ice cream, frozen yogurt, and sherbet
Low or nonfat potato chips, tortilla chips, corn chips and pretzels
Low-fat Butter (Cereals and Breads)
Low-fat Butter (Fats on potatoes, rice, noodles and beans)
Low-fat Butter (Fats used on vegetables)
Lowfat cottage cheese and ricotta cheese
Margarine, lowfat (Cereals and Breads)
Margarine, lowfat (Fat used in cooking)
Margarine, lowfat (Fats on potatoes, rice, noodles and beans)
Margarine, lowfat (Fats used on vegetables)
Margarine, stick (Cereals and Breads)
Margarine, stick (Fat used in cooking)
Margarine, stick (Fats on potatoes, rice, noodles and beans)
Margarine, stick (Fats used on vegetables)
Margarine, tub (Cereals and Breads)
Margarine, tub (Fat used in cooking)
Margarine, tub (Fats on potatoes, rice, noodles and beans)
Margarine, tub (Fats used on vegetables)
Mayonnaise, fat free or nonfat
Mayonnaise, low or reduced fat
Mayonnaise, regular
Meal replacement drinks and shakes containing soy or soy protein [SOY]
Meal replacement drinks and shakes such as Slim-Fast, Ensure and Carnation Instant Breakfast
Meat gravies
Miso soup
Muffins, scones, croissants and biscuits
Nonfat cheese and nonfat cream cheese, including cheese used in cooking
Nonfat cottage cheese and ricotta cheese
Nonfat sour cream (Fats on potatoes, rice, noodles and beans)
Nonfat sour cream (Fats used on vegetables)
Noodles and other grains (as a side dish)
Other candy, such as Lifesavers, licorice and jelly beans
Other fortified cereals
Other soups such as chicken noodle
Other soy desserts such as cheesecake
Packaged mixed dishes with soy or tofu
Pancakes, French toast, and waffles
Pizza
Plain popcorn (no butter) or lowfat microwave popcorn
Potato, macaroni and pasta salads made with mayonnaise or oil
Pudding, custard and flan
Refried beans
Regular cottage cheese and ricotta cheese

Regular crackers, such as Ritz and Wheat Thins
Regular potato, tortilla chips, corn chips and puffs
Regular sour cream (Fats on potatoes, rice, noodles and beans)
Regular sour cream (Fats used on vegetables)
Salad dressing - fat free
Salad dressing - lowfat
Salad dressing - regular
Soy cheese
Soy crackers
Soy ice cream
Soy sauce, tamari, teriyaki sauce, Szechwan sauce and natto
Spaghetti and other pasta with oil, cheese or cream sauce, including macaroni and cheese
Spaghetti, lasagna and other pasta with tomato sauce (and no meat)
Sports bar and meal replacement bar containing soy
Sports or meal replacement bars such as Power Bars and Cliff Bars
Standard cold cereals
Sweetened cold cereals
Tofu or soy breakfast sausage or other breakfast meat
Tofu or soy chicken or turkey
Tofu or soy cold cuts, hot dogs or deli substitute
Vegetable, minestrone and tomato soup
Veggie soy or tofu burgers or ground meat substitute
White breads, including bagels, rolls and English muffins
Yogurt, sweetened or with fruit, full-fat
Yogurt, sweetened or with fruit, lowfat
Yogurt, sweetened or with fruit, nonfat
**FFQ foods that are UPF but accounted for elsewhere in score and not included in aUPF food list**
Counted toward red and processed meat intake:
All other lunch meat such as bologna, salami and Spam
Bacon and breakfast sausage
Low or reduced fat hot dogs and sausage
Lunch meats such as ham, turkey and lowfat bologna
Regular hot dogs and sausage such as bratwurst and chorizo
Spaghetti, lasagna and other pasta with meat sauce
Stew, pot pie, curries and casseroles with meat or chicken
Counted toward sugar-sweetened beverage intake:
Fruit drinks fortified with Vitamin C, such as Hi-C, Fruitopia, and Kool-Aid
Lattes
Regular soft drinks (not diet)
Sweet tea and bottled sweetened tea drinks
Counted toward alcohol intake:
Liquor and mixed drinks
Counted as high-fiber food:

Complete or primarily whole grain cold cereal
Cooked whole grain cereals
Lowfat whole grain crackers
Regular whole grain crackers
Spaghetti and other pasta with oil, cheese or cream sauce, including macaroni and cheese (whole wheat)
Spaghetti, lasagna and other pasta with tomato sauce (whole wheat and no meat)
Spaghetti, lasagna and other pastas with meat sauce (whole wheat)
Whole grain breads, including bagels and rolls
Whole grain breads, including bagels and rolls (100% Whole Grains)

[a]UPF classification based upon NOVA standards and collaboration with investigators at NCI and AICR.
